# Very rare malposition of central venous catheter in cardiac surgery patients

**DOI:** 10.5830/CVJA-2022-062

**Published:** 2023-02-06

**Authors:** Nursen Tanrikulu, Ali Haspolat, Ali Sefik Koprulu

**Affiliations:** Department of Anesthesiology and Reanimation, Kolan International Hospital, Istanbul, Turkey; Department of Anaesthesiology and Reanimation, Yeni Yüzyıl University Medical Faculty, Istanbul, Turkey

**Keywords:** malposition, left internal mammary vein, central venous catheter, complications

## Abstract

Malposition of a catheter is found in approximately 7% of cases after central venous catheterisation. This may result in haemorrhage, venous thrombosis and functional impairment, depending on the injury to the vessel wall. Uncomplicated catheterisation, easy aspiration of blood and monitoring of catheterisation do not guarantee correct placement of the catheter. In our rare case series, we share our experience of four cases of malposition into the left internal mammary vein (LIMV) that we experienced in a three-year period. The thinness and fragility of the vessel wall, particularly, increases the probability of complications in malposition into the LIMV. Administration of a catheter through the right jugular vein is associated with the lowest incidence of malposition. Performing the procedure under the guidance of ultrasonography (USG) and confirmation of the catheter position after puncture using one of the USG techniques will minimise the probability of malposition. In addition, a lung X-ray should immediately be taken, and venography and fluoroscopy should be considered in the presence of suspicion.

Open-heart surgery is one of the procedures in which central venous catheterisation (CVC) is routinely performed. Although it is a simple and safe intervention, various complications may occur during and after the procedure, even when performed by the most experienced surgeons. The rate of these complications is estimated to be approximately 20%, alhough it varies in different studies.^1^

Successful catheterisation requires not only technical expertise but also awareness of potential complications and timely intervention. One of the frequently seen complications in the early term is malposition.^2^ The tip of the catheter ideally should be placed out of the pericardial sac and as wide as possible, parallel to the long axis of a central vein.^3^ The vessel most used in cardiac surgery is the superior vena cava (SVC).

In this study, we aimed to increase the awareness of such complications and emphasise possible action to be taken to minimise complications by sharing our experience of four cases of malposition of the central venous catheter inserted into the left internal mammary vein (LIMV) during coronary artery bypass grafting (CABG) surgery performed between 1 January 2018 and 31 December 2020.

## Case report 1

A 61-year-old male patient scheduled for CABG had an uncomplicated puncture through the right internal jugular vein (RIJV) after anaesthetic induction. The guide-wire could not be forwarded further than 10 cm.

**Fig. 1:**
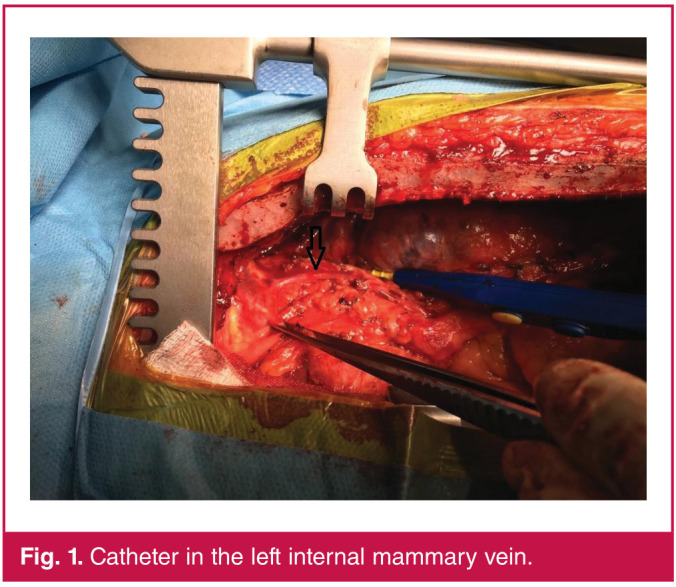
Catheter in the left internal mammary vein.

So as not to put the patient, who had partial stenosis in the right carotid artery, at risk, the left side was attempted. A triple-lumen 7-F catheter was inserted through the LIJV without complication. Free flow of blood was confirmed through all three ports and the catheter was fixed at 16 cm. The central venous pressure (CVP) was high (16–17 cm H2O) and the trace was irregular. During dissection of the left internal mammary artery (LIMA), the surgical team found the catheter to be inserted into the LIMV (Fig. 1). No rupture or haemorrhage was present and vascular integrity was intact. However, leakage of fluid out of the catheter was observed after treatment with pressurised fluid. The catheter was withdrawn and repositioned intra-operatively. The operation was continued with a CVP of 8 cm H2O. No other peri-operative or postoperative complication was found.

## Case report 2

LIJV catheterisation was performed after anaesthetic induction since cannulation of the RIJV was secheduled for cardiopulmonary bypass in a 64-year-old male patient who would undergo minimal invasive CABG through a left anterior thoracotomy. The CVP trace could not be monitored although blood could be aspirated easily through the catheter and the CVP was inconsistent. The catheter was found to be malpositioned in the LIMV by the surgeon during dissection of the LIMA. The catheter was withdrawn under surgical supervision and catheterisation was repeated.

## Case report 3

The LIJV catheterisation procedure was completed without complications in a 57-year-old female patient since the cardiovascular anaesthesiologist was highly experienced in paediatric SVC cases. The trace was monitored, but the CVP was low. The patient was haemodynamically stable. The surgeon found the catheter in the LIMV during dissection of the LIMA. The vessel lumen was intact and no complication was encountered. After completion of dissection of the LIMA, the catheter was repositioned safely into the innominate vein. CABG was completed without additional complications.

## Case report 4

The LIJV was used for catheterisation in a 61-year-old male patient scheduled for cannulation of the right subclavian vein prior to cardiopulmonary bypass. A high CVP of 24–25 cm H2O was measured in the patient and free blood aspiration was achieved from all the lumens of the catheter. The high CVP was considered to be secondary to moderate portal hypertension detected in the pre-operative period and the patient was also found to be partially dehydrated. The surgeon found the catheter in the LIMV during dissection of the LIMA. The catheter was withdrawn by the surgeon and repositioned under surgical supervision.

## Discussion

The CVC procedure is one of the most frequently performed invasive interventions, not only in anaesthesiology and intensive care units but also in various specialisations from oncology to emergency medicine. This widespread use has resulted in undesirable consequences, such as the perception that complications are normal and acceptable.^1^ Although complication rates are varied in the literature, the rates of infection, haematoma and pneumothorax are reported at 5–26, 2–26 and 30%, respectively.^2^ Another less common complication with CVC is malposition of the catheter tip in an incorrect location and it has been reported in approximately 7% of cases.^2^

The causal mechanism of malposition is multifactorial and not entirely clear. Some studies have shown that direction of slope of the needle facilitates the orientation of the guide-wire to the desired target.^4^ On the other hand, some researchers have advocated that structural properties of the body such as obesity and extremely large chest size increase the risk for malposition.^5^

In fact, an approximately 9-mm displacement of the catheter tip has been demonstrated during breathing, particularly expiration.^6^ Other researchers have stated that misorientation depends on variations in the venous anatomy. These variations lead to malposition by causing misorientation of the catheter tip towards the low-resistance aberrant vessel arms. Venous malformation may be congenital or acquired.^7^

Congenital variations are usually asymptomatic and can be detected radiologically, which is frequently performed for control after catheterisation. They may complicate radiological detection of the accurate localisation of the catheter tip. The most common congenital variation is persistent left vena cava. It is encountered in 0.3 and 4.3% of healthy patients and those with congenital heart disease, respectively.^8^ No congenital vascular anomalies were found in our case series.

In contrast to the IMV normally originating from the subclavian artery, the IMV may originate from the internal innominate vein. It should be borne in mind that different variations of the IMV may be present.^9^ The entry orifice of the LIMV is more distant from the veins on the right side, therefore this vein can be reached only by a catheter coming through the left brachiocephalic vein. In patients with portal hypertension, increased blood pressure may enlarge the IMV from the collateral circulation.^10^ Consequently, that increases the risk for malposition of the catheter. Liminal portal hypertension was present in our fourth patient and that may be one of the possible causes of the malposition.

Acquired malformations are more commonly seen than congenital variations. Malformations may have an internal or external origin.^11^ More than 85% of the vascular distortions with an external origin are associated with malignancy (lung or breast cancer, lymphoma, germ cell tumour). Benign factors include substernal goiter, thymoma, cystic hygroma and histoplasmosis. Atelectasis and pleural fluid can also push or pull venous structures away from the midline. Internal factors are more commonly vascular thrombosis and stenosis. Recent surgical treatment, malignancy, immobilisation, haemodialysis, chemotherapy and pregnancy increase the risk for thrombosis. Likewise, excessive use of a vessel, subclavian catheterisation and interventions performed from the left side of the neck may cause frequent vascular stenosis.^12^ None of these factors was found in our patients.

Malposition with CVC occurs much more frequently after procedures performed in the left side of the neck (internal jugular or subclavian vein). Schummer et al. carried out a prospective study involving 1 794 catheterisations performed by experienced practitioners and found a malposition rate of 6.7%. The malposition rates for the LIJV, right subclavian vein, left subclavian vein and RIJV were 12, 9.3, 7.3 and 4.3%, respectively.^13^ It has also been found in other studies that malposition in left-sided catheterisation is higher than in those performed from the right side.^14^ The higher malposition rate in interventions performed from the left side is because of the much longer left brachiocephalic vein compared with the right brachiocephalic vein, more oblique position of the heart and greater number of small vascular branchings in that region.^15^ In addition, there are two perpendicular angles en route from the LIJV to CVC.^3^

The intervention was performed through the LIJV due to mandatory reasons in two patients in our case series. Under normal circumstances, the right side would primarily be considered for CVC in the absence of a contra-indication. Besides, it was found in the study by Marik et al. involving 113 652 catheter days that there was no difference between femoral, subclavian and internal jugular interventions regarding catheter-related haematogenous reproductions, in contrast to general belief.^16^ The length of the catheter should also be adjusted according to the selected localisation. Incorrect length of catheter increases the probability of malposition or displacement of the catheter through the vein via migration.^17^

The commonly used method after catheterisation for detection of localisation and complications is anterior–posterior lung radiography. However, this is postponed to the postoperative period in routine clinical practice if no complication has been experienced with regard to puncture and forwarding of the pre-operatively inserted catheter.

Use of ultrasonography (USG) during catheterisation facilitates accurate identification of the veins and puncture site, however, it does not prevent malposition.^18^ Some authors recommend use of USG to confirm the accurate intravascular position of the catheter and detect possible complications early on.^19^,^20^ Bedside USG has some practical benefits to the conventional radiological examination, such as much faster application, more comfortable to use in different environments and protection of patients against radiation exposure. Researchers recommend the use of USG not only during puncture, but also for accurate orientation of the guide-wire by visualising it during the rest of the procedure.^21^

In some studies, echocardiography has been used during insertion of the guide-wire. The guide-wire was forwarded and localised as a hyperechogenic line in the right atrium for confirmation of the position, then it was withdrawn under control until its ‘J’ tip disappeared into the right atrium and was fixed in the correct position.^21^,^22^ In addition, CVC can be visualised through the right supraclavicular fossa and forwarding of the guide-wire towards this field after puncture can easily be monitored and its localisation can be confirmed.^23^

According to a meta-analysis, the best confirmation of the location of CVC can be achieved by a combination of vascular USG and a transthoracic echocardiogram.^20^,^24^ Briefly, it is possible to reduce the malposition rate to zero by implementing certain protocols. In our clinic we now use USG mandatorily for puncture and forwarding of the guide-wire in catheterisations performed in the operating theatre and intensive care unit.

We also perform anterior–posterior lung radiography after completion of the procedure. In the case of suspected catheter malposition, we perform lateral radiograpy in addition to these routine studies and also venography using a little diluted radiopaque agent. Furthermore, we carry out imaging with C-armed endoscopy in the operating theatre in case of ongoing suspicion.^25^

Localisation of the CVC tip in a vessel other than the SVC may result in consequences such as haemorrhage due to abrasion or rupture of the vessel wall, local venous thrombosis, dysfunction of the catheter due to folding in on itself, and entry of the administered drugs to the cerebral circulation by cranial retrograde injection instead of the systemic circulation.^2^ Localisation of the catheter in a small vessel increases the risks.^26^

Infusion of hyperosmolar solutions or vasopressors through the catheter, as is frequently performed in cardiovascular surgery, increases the possibility of complications.^2^ Besides, the localisation of the catheter in the atrium instead of a side-vessel branch may cause arrhythmia or result in atrial perforation.^27^ The use of this route should not be attempted intentionally. Early detection and repositioning of the malpositioned central venous catheter may prevent serious complications.

We experienced no serious complications in our cases since the malposition was detected early in the intra-operative stage. However, probable malposition could not be detected if dissection of the LIMA was not performed since no difficulty was experienced during catheterisation and blood could easily be aspirated. Consequently the patient could be subject to complications. The only clinical symptom reported in the literature of other IMV malpositions was development of chest and/or shoulder pain due to administration of fluid.^28^,^29^ In all our cases, clinical symptoms could not be evaluated since catheterisation was performed after induction of anaesthesia. However, no complication was experienced during puncture or forwarding of the catheter and our experienced practitioner described the intervention as an ordinary procedure. CVP of only two of our cases was not correlated with clinical results.

## Conclusion

Central venous catheter positioning should be considered as a procedure that may develop complications, even when performed by the most experienced practitioners. Easy aspiration of blood through the catheter and monitoring the CVP trace do not guarantee the correct position of the catheter. Since the central venous catheter is inserted after anaesthetic induction in cardiovascular surgery, no clinical symptoms can be detected in a case of malposition. The probability of complications is high because of the fragility of particularly the IMV walls. In the case of unnoticed malposition during the operation, peri/postoperative CVP will be inaccurate and it will lead to misdiagnosis and false treatment in cases of rupture.

Our suggestion is the implementation of central catheterisation through the RIJV, which produces the lowest complication rates, and under the guidance of bedside USG as an easily applicable non-invasive method to confirm the position after puncture. Additionally, lung radiography should be performed immediately after the procedure, and lateral radiography and venography also should be carried out in the case of suspicion related to positioning of the catheter.
